# Pharmacological rescue of specific long QT variants of KCNQ1/KCNE1 channels

**DOI:** 10.3389/fphys.2022.902224

**Published:** 2022-11-23

**Authors:** Xinle Zou, Xiaoan Wu, Kevin J. Sampson, Henry M. Colecraft, H. Peter Larsson, Robert S. Kass

**Affiliations:** ^1^ Department of Molecular Pharmacology & Therapeutics, Vagelos College of Physicians & Surgeons of Columbia University Irving Medical Center, New York, NY, United States; ^2^ Department of Physiology and Biophysics, Miller School of Medicine, University of Miami, Miami, FL, United States

**Keywords:** long QT syndrome, IKS, potassium channel, pharmacology, KCNQ1, KCNE1

## Abstract

The congenital Long QT Syndrome (LQTS) is an inherited disorder in which cardiac ventricular repolarization is delayed and predisposes patients to cardiac arrhythmias and sudden cardiac death. LQT1 and LQT5 are LQTS variants caused by mutations in KCNQ1 or KCNE1 genes respectively. KCNQ1 and KCNE1 co-assemble to form critical I_KS_ potassium channels. Beta-blockers are the standard of care for the treatment of LQT1, however, doing so based on mechanisms other than correcting the loss-of-function of K^+^ channels. ML277 and R-L3 are compounds that enhance I_KS_ channels and slow channel deactivation in a manner that is dependent on the stoichiometry of KCNE1 subunits in the assembled channels. In this paper, we used expression of I_KS_ channels in Chinese hamster ovary (CHO) cells and *Xenopus* oocytes to study the potential of these two drugs (ML277 and R-L3) for the rescue of LQT1 and LQT5 mutant channels. We focused on the LQT1 mutation KCNQ1-S546L, and two LQT5 mutations, KCNE1-L51H and KCNE1-G52R. We found ML277 and R-L3 potentiated homozygote LQTS mutations in the I_KS_ complexes-KCNE1-G52R and KCNE1-L51H and in heterogeneous I_KS_ channel complexes which mimic heterogeneous expression of mutations in patients. ML277 and R-L3 increased the mutant I_KS_ current amplitude and slowed current deactivation, but not in wild type (WT) I_KS_. We obtained similar results in the LQT1 mutant (KCNQ1 S546L/KCNE1) with ML277 and R-L3. ML277 and R-L3 had a similar effect on the LQT1 and LQT5 mutants, however, ML277 was more effective than R-L3 in this modulation. Importantly we found that not all LQT5 mutants expressed with KCNQ1 resulted in channels that are potentiated by these drugs as the KCNE1 mutant D76N inhibited drug action when expressed with KCNQ1. Thus, our work shows that by directly studying the treatment of LQT1 and LQT5 mutations with ML277 and R-L3, we will understand the potential utility of these activators as options in specific LQTS therapeutics.

## Introduction

The congenital Long QT Syndrome (LQTS) is a hereditary cardiac disease characterized by a prolongation of the QT interval (QTc) on the electrocardiogram (ECG). This disease can lead to arrhythmias and sudden cardiac death and can be either a rare autosomal recessive form called Jervell and Lange-Nielsen syndrome (J-LN), which is associated with deafness (Jervell and Lange-Nielsen, 1957), or more commonly an autosomal dominant form known as Romano-Ward syndrome (R-W), which is not associated with deafness (Ward, 1964). The estimated prevalence of congenital LQTS is approximately at least 1 in 2,500 (Schwartz et al., 2009). The prevalence of LQTS mutations may be even higher than reported because up to 36% of genotype confirmed LQTS patients have normal QTc (Priori et al., 2003; Barsheshet et al., 2011; Goldenberg et al., 2011). Congenital LQTS is subdivided, to date, into 16 types based on distinct genes in which causative mutations occur (Bohnen et al., 2017; Giudicessi et al., 2018).

KCNQ1 (I_KS_ channel *α* subunits, KvLQT1, Kv7.1) and KCNE1 (I_KS_ channel *β* subunit) assemble to form the slow delayed rectifier I_KS_ potassium channels in the heart (Barhanin et al., 1996; Sanguinetti et al., 1996). Disease causing mutation in KCNQ1 is referred to as LQT-1 whereas mutations in KCNE1 are characterized as LQT-5; in both cases the mutations cause a loss of I_KS_ channel function (Bohnen et al., 2017). Taken together, mutations in LQT1 and LQT5 comprise nearly 50% of all congenital LQTS patients (Splawski et al., 2000; Tester et al., 2005; Kapplinger et al., 2009; Lieve et al., 2013). LQT-1 patients have been shown to be at an increased risk of arrhythmias and/or sudden cardiac death during exercise-induced elevated sympathetic nerve activity (Schwartz et al., 2001), and both LQT-1 and LQT-5 are associated with arrhythmias risk and sudden cardiac death across the entire age spectrum from infancy to adults (Giudicessi et al., 2018). Not surprisingly, beta-blockers are the standard of care for the treatment of LQT1 and have been shown to reduce recurrent syncope and mortality in LQT1 patients, however through reduction in sympathetic activity in the heart and not by correcting the loss-of-function of K^+^ channels (Moss et al., 2000; Gemma et al., 2011). Until now, genotype-specific management and treatment have yet to be achieved for patients with mutations in the I_KS_ channel complex.

Evidence has been presented that KCNQ1 and KCNE1 subunits together form the KCNQ1/KCNE1 channel with KCNQ1 to KCNE1 stoichiometries ranging from 4:1 to 4:4 in heterologous expression systems (Wang et al., 1998; Nakajo et al., 2010), and embryonic stem cell myocytes (Wang et al., 2011; Wang and Kass, 2012). The exact stoichiometry of assembled physiologically relevant channels remains unknown as some lines of evidence point toward 4:2 (Morin and Kobertz, 2008; Plant et al., 2014) while others including the cryo-EM structure of KCNQ1/KCNE3 (a paralog of KCNE1) supports the idea that a 4:4 stoichiometry (Sun and MacKinnon, 2020). It is generally accepted that the ratio of KCNE1 to KCNQ1 subunits in I_KS_ channels is flexible (Murray et al., 2016; Fedida, 2018; Wang et al., 2020a; Wang et al., 2020b).

ML277 is a compound that has been identified as a specific and potent activator of KCNQ1 channels that acts with a concomitant slowing of channel deactivation (Yu et al., 2010; Mattmann et al., 2012; Yu et al., 2013). Evidence has been presented that KCNQ1 and KCNE1 subunits together form the KCNQ1/KCNE1 channel with KCNQ1 to KCNE1 stoichiometries ranging from 4:1 to 4:4 in heterologous expression systems (Wang et al., 1998; Nakajo et al., 2010), and embryonic stem cell myocytes (Wang et al., 2011; Wang and Kass, 2012). The dynamic stoichiometry of I_KS_ subunits confers a progressive continuum of pharmacological sensitivity of the expressed channels (Yu et al., 2013). For example, an increase in the number of *β* subunits per *α* subunit reduces the effect of ML277 (Nakajo et al., 2010; Yu et al., 2013). Like ML277, the benzodiazepine derivative R-L3 also selectively increases KCNQ1 channel current amplitude with a slowing of channel deactivation (Salata et al., 1998; Yu et al., 2013).

Because of the important role of the assembly of KCNE1 subunits in the response of KCNQ1/KCNE1 channels to these pharmacological agents, we sought to determine whether these compounds might have distinct potential in rescuing the activity of KCNQ1 channels assembled with KCNE1 subunits harboring specific LQT-1 and LQT-5 mutations that have previously been shown to affect the I_KS_ complex assembly and thus altering modulation of KCNE1-bound channels: KCNE1-L51H (Bianchi et al., 1999), KCNE1-G52R (Ma et al., 2003) and KCNQ1 S546L (Dvir et al., 2014). The results of our study provide evidence that the I_KS_ activators ML277 and R-L3 do in fact have potential utility to restore I_KS_ channel activity for these LQT-1 and LQT-5 mutant channels.

## Materials and methods

### Constructs and mutagenesis

The wild type of human KCNQ1 was cloned in the pcDNA3.1 (+) vector. The Quick-Change Site-Directed Mutagenesis Kit (Agilent Technologies, Santa Clara, CA) was used to prepare KCNQ1-S546L, KCNQ1-T265I, KCNE1-L51H, KCNE1-G52R, KCNE1-G55C, and KCNQ1-D76N mutants according to the manual. Sequences of the entire open reading frame of mutated KCNQ1 or KCNE1 were confirmed.

### cRNA preparations and *Xenopus* oocyte injection

cRNAs were prepared by first linearizing the DNA plasmid using NheI-HF followed by *in vitro* RNA transcription using the mMessage mMachine T7 kit (Ambion, Austin, TX). The concentration of cRNA was determined by absorbance at 260 nm. The pGEM-HE vector was used for *Xenopus* oocyte expression. A total of 50 ng of KCNQ1 cRNA was injected into defolliculated *Xenopus laevis oocytes* (Ecocyte, Bioscience, Austin, TX) using a Drummond injector (Broomall, PA). Oocytes were incubated at 16°C for ∼36–48 h before recordings.

### Two electrode voltage clamp recordings

For KCNQ1/KCNE1 experiments, oocytes were co-injected with KCNQ1 cRNA and KCNE1 cRNA by a ratio of 3:1, weight:weight. After cRNA injection, oocytes were incubated in ND96 solution (96 mM NaCl, 2 mM KCl, 1 mM MgCl_2_, 1.8 mM CaCl_2_, 5 mM HEPES; pH = 7.5) for 2–5 days before electrophysical experiments. Oocytes were recorded in ND96 solution in a two-electrode voltage clamp configuration using a DAGAN CA-1B Two-electrode voltage-clamp amplifier (Dagan Corporation, MN, United States) and a Axon Digidata 1550B acquisition system (Molecular Devices, San Jose CA). Microelectrodes were pulled to resistances from 0.5 to 1 MΩ and filled with 3M KCl. Electrophysiological recordings were obtained using Clampex 10.3 software (Pclamp 10, Molecular Devices, San Jose, CA). For cadmium experiments, voltage steps from −80 mV to +40 mV followed by a tail voltage of −40 mV. The blockade of channels by cadmium was measured at the end of the +40 mV test pulse using Clampfit 10.3 software (Pclamp 10, Molecular Devices, San Jose, CA).

Oocytes were placed in a recording chamber filled and perfused with ND96 solution (96 mM NaCl, 2mM KCl, 5 mM HEPES, 1.8 mM CaCl_2_, and 1 mM MgCl_2_, pH7.5). 100 µM LaCl_3_ was used to block the endogenous currents in the oocyte. The oocytes were held at −80 mV. KCNQ1-KCNE1 currents were evoked by depolarizing the oocyte to +40 mV for 5 s and then repolarizing to −40 mV for 3 s and were recorded every 15 s to monitor the effect of 0.5 mM Cd^2+^.

### Cell culture and transfection

Low-passage-number Chinese hamster ovary (CHO) cells (American Type Culture Collection) were cultured at 37°C in Ham’s F12 medium with 10% fetal bovine serum (FBS) and 100 μg/ml of penicillin-streptomycin. Cells were transiently transfected with desired plasmids including KCNQ1, KCNE1, and their mutants in a 25 cm^2^ flask. Lipofectamine and Plus reagent (Invitrogen) were used for transfection when cells reached 20–30% confluence.

### Whole-cell patch clamp recordings

Cells were plated in 3.5 cm culture dishes on the stage of an inverted microscope (OLYMPUS BH2-HLSH, Precision Micro Inc., Massapequa, NY). Currents were recorded at room temperature using the whole-cell patch-clamp technique (series resistance was 1.5–3 MΩ) by an Axopatch 200B amplifier (Axon Instruments, Foster City, CA). Patch-clamp protocols have been described previously (Terrenoire et al., 2009). The voltage was stepped to +60 mV for 2 s from a −70 mV holding potential and then followed by a 2 s repolarizing pulse to −40 mV during which I_KS_ tail current was measured. This pulse sequence was applied at a repetition interval of 10 s. External solutions contained the following: 132 mM NaCl, 4.8 mM KCl, 2 mM CaCl_2_, 1.2 mM MgCl_2_, 10 mM HEPES and 5 mM glucose (pH was adjusted to 7.4 with NaOH). Internal solution contained the following: 110 mM KCl, 5 mM ATP-K_2_, 11 mM EGTA, 10 mM HEPES, 1 mM CaCl_2_ and 1 mM MgCl_2_ (pH was adjusted to 7.3 with KOH). Currents were sampled at 10 kHz and filtered at 5 kHz. In all experiments we waited 1–2 min until channel activity was stable and not running down. Drugs were then applied and channel activity in the presence of drug was then measured after channel activity stabilized.

### Data analysis

**FIGURE 1:**
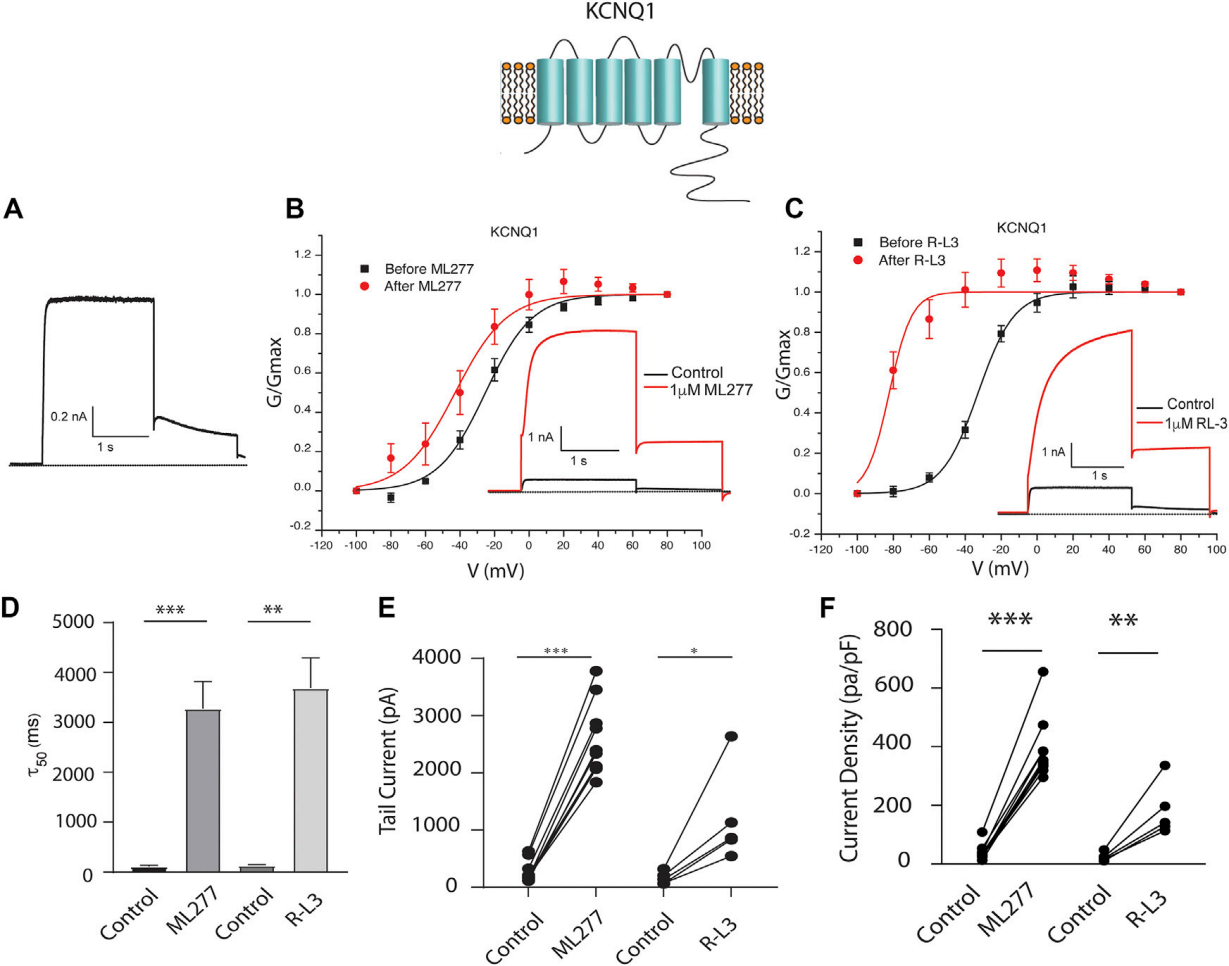
ML277 and R-L3 modulate wild type IKS channels in the absence of KCNE1. Individual current traces recorded in response to 2 s depolarizing steps to +60 mV from **(A)** 70 mV holding potential are shown as insets in panels **(B,C)**. Currents are shown in the absence (black traces) and presence (red traces) of test drugs. **(A)**. Control drug-free recording of wild type KCNQ1 channel activity. **(B)**. KCNQ1 conductance-/voltage relationships recorded in the absence (black) and presence (red) of 1 μM ML277. **(C)**. KCNQ1 conductance-/voltage relationships recorded in the absence (black) and presence (red) 1 μM R-L3. **(D)**. Deactivation tail time constants measured at −40 mV in the absence (control) and presence of 1 μM ML277 and 1 μM R-L3. **(E)**. KCNQ1 tail current amplitude (measured at −40 mV) in the absence (control) and presence of ML277 (1 μM) and R-L3 (1 μM). **(F)**. Current density measured at +60 mV for the conditions indicated on the abscissa. Data from individual experiments in **(E,F)** are connected by straight lines.

Patch-clamp data, shown as mean ± S.E., were acquired using pCLAMP 8.0 (Axon Instruments) and analyzed with Origin 7.0 (Origin Lab, Northampton, MA) and Clamp fit 8.2 (Axon Instruments). Statistical data analysis was assessed with Student’s t-test for simple comparisons was carried out using Excel. Data points were taken before and after drug and were time-matched following stable responses. In the figures, statistically significant differences determined using student’s t-test to control values which are marked by asterisks (*, *p* < 0.05; **, *p* < 0.01; ***, *p* < 0.001) are indicated in figures. Paired t-tests were applied for data in figures 1 through 9. For oocyte data, all experiments were repeated more than three times from at least two batches of oocytes. All experiments were repeated more than three times from at least two batches of oocytes. Statistical data analysis was performed using Student’s *t*-test or ANOVA with a Dunnett’s test. Data are presented as mean ± SEM, and *n* represents the number of experiments.

## Results

### ML277 and R-L3 enhance wt I_KS_ channel activity in the absence but not in the presence of KCNE1

**FIGURE 2:**
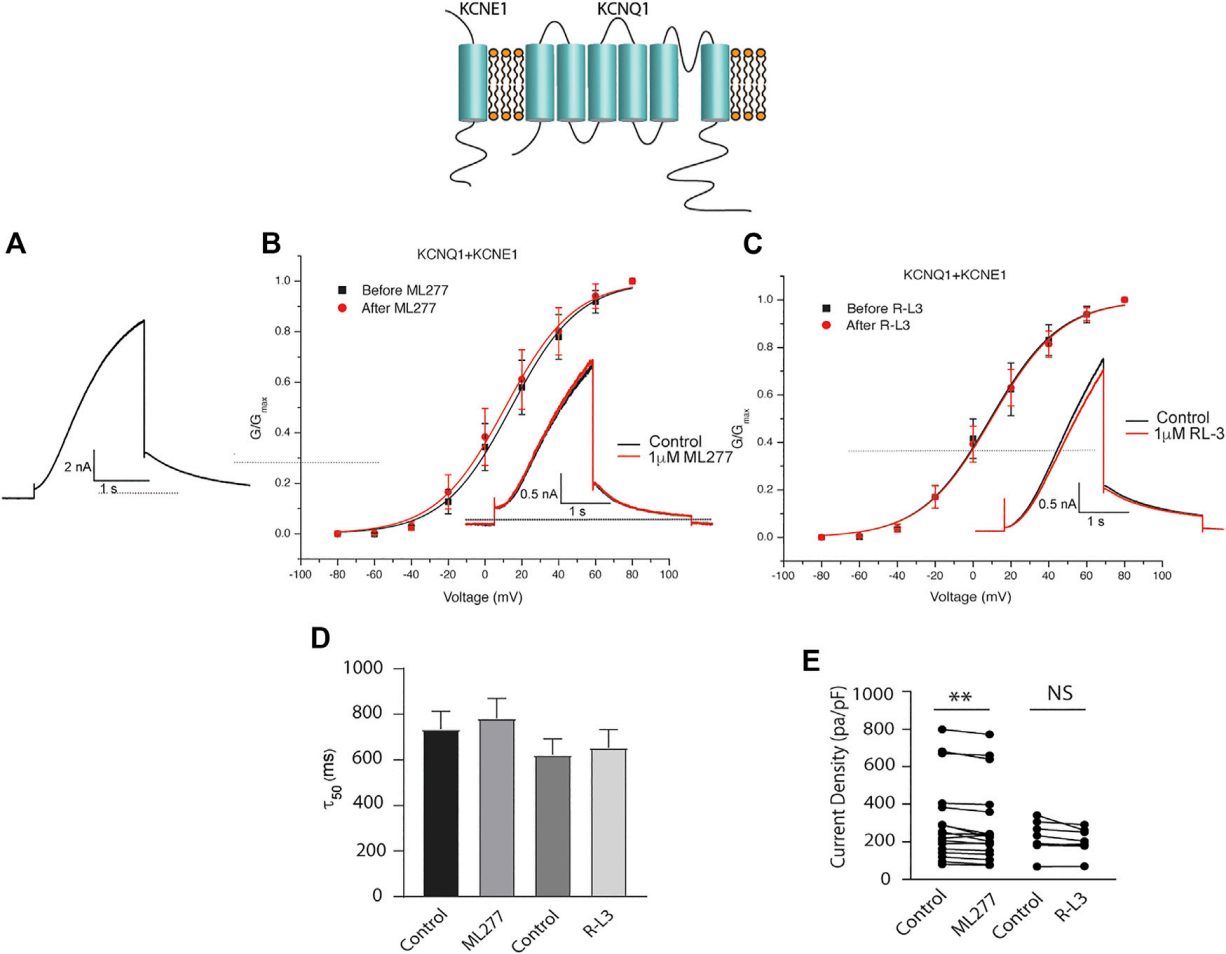
ML277 and R-L3 do not modulate IKS channels in the presence of KCNE1. Individual current traces recorded in response to 2 s depolarizing steps to +60 mV from **(A)** 70 mV holding potential are shown as insets in panels **(B,C)** in the absence (black) and presence (red) of test drugs. **(A)**. Control KCNQ1-KCNE1 channel activity recorded at +60 mV. **(B)**. KCNQ1-KNCE1 conductance-voltage curves in the absence (black) and presence (red) of 1 μM ML277. **(C)**. KCNQ1-KNCE1 conductance-voltage curves in the absence (black) and presence (red) of 1 μM R-L3. **(D)**. Deactivation tail time constants measured at −40 mV in the absence (control) and presence of 1 μM ML277 and 1 μM R-L3. **(E)**. Summary data for peak KCNQ1-KCNE1 current density measured at +60 mV in the absence (control) and presence of 1 μM ML277 and 1 μM R-L3. Data from individual experiments are connected by straight lines.

To test our hypothesis that ML277 and R-L3 might serve as therapeutic agents in the treatment of some LQT-1 and LQT-5 mutation carriers, we first confirmed the efficacy of these compounds on I_KS_ channels expressed in the absence and presence of KCNE1 subunits. We expressed KCNQ1 channels in the absence of KCNE1 (KCNE1:KCNQ1 = 0:1) and then in the presence of KCNE1, using equal amounts of subunit cDNA (KCNE1:KCNQ1 = 1:1). As illustrated in Figure 1, we confirm that both ML277 and R-L3 markedly enhance I_KS_ channel activity and slow channel deactivation in the absence of KCNE1 as has been previously reported (Salata et al., 1998; Yu et al., 2013). Further, expression of KCNE1 with KCNQ1 inhibits drug activity yielding channels that are insensitive to 1 μM ML277 or R-L3 (Figure 2), again consistent with previous reports (Salata et al., 1998; Yu et al., 2013). These data establish in our experiments that ML277 and R-L3 both significantly potentiate I_KS_ channels in the absence, but not in the presence, of KCNE1 and serve as controls for our experiments testing the effects of these compounds on channel assembled with specific LQT-5 and LQT-1 mutations.

### ML277 and R-L3 rescue selected LQT5 I_KS_ currents

**FIGURE 3:**
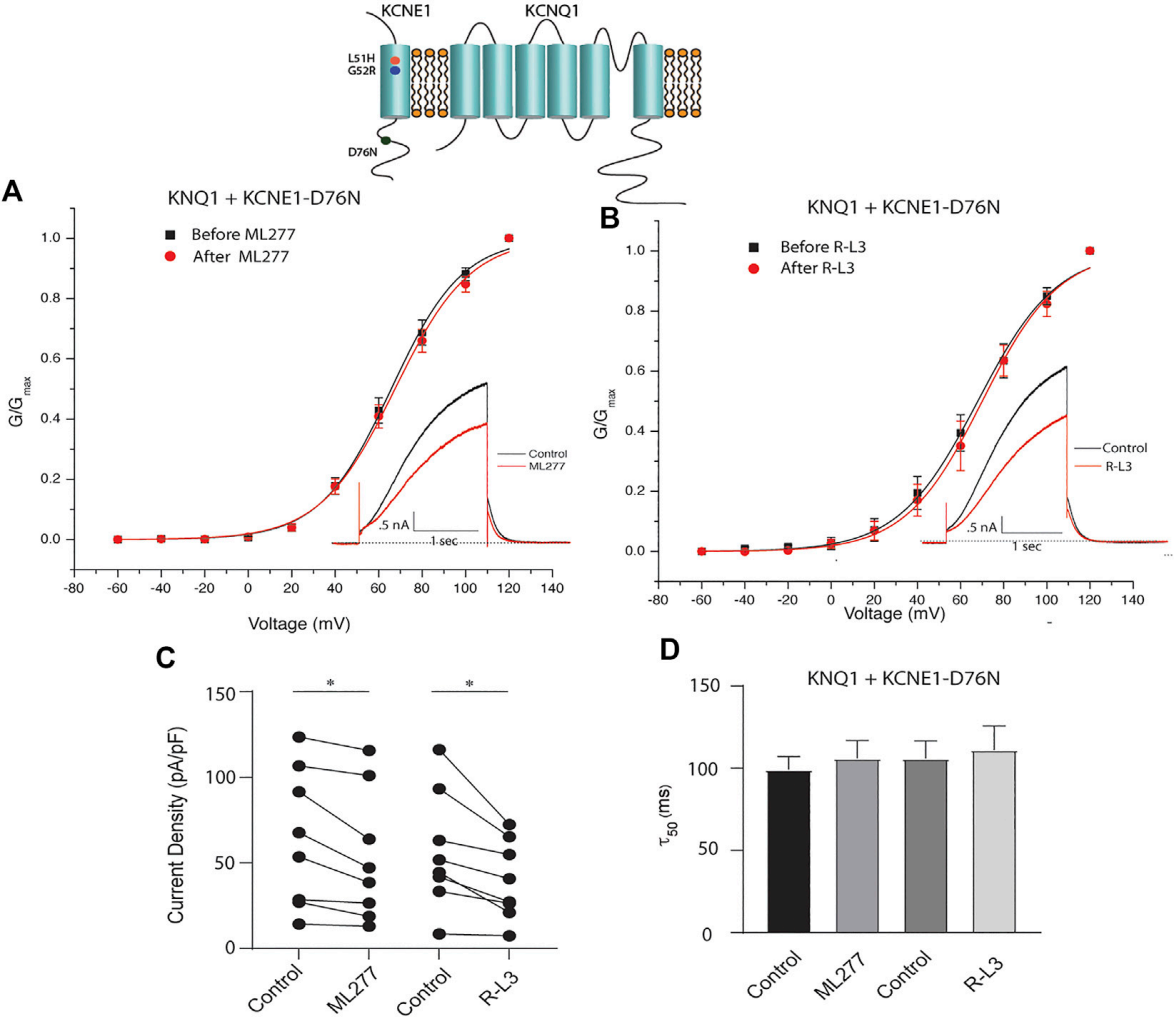
ML277 and R-L3 do not enhance channel activity carried by KCNQ1 expressed with the D76N mutant KCNE1 subunit. **(A)**. Conductance voltage curves of KCNQ1-D76N channels in the absence (black) and presence (red) of 1 μM ML277. **(B)**. Conductance voltage curves of KCNQ1-D76N channels in the absence (black) and presence (red) of 1 μMR-L3. **(C)**. Tail current density measured at −40 mV in the absence (control) and presence of 1 μM ML277 and 1 μM R-L3. Data from individual experiments are connected by straight lines. **(D)**. Deactivation time constants measured at −40 mV in the absence (control) and presence of 1 μM ML 277 and 1 μM R-L3.

**FIGURE 4:**
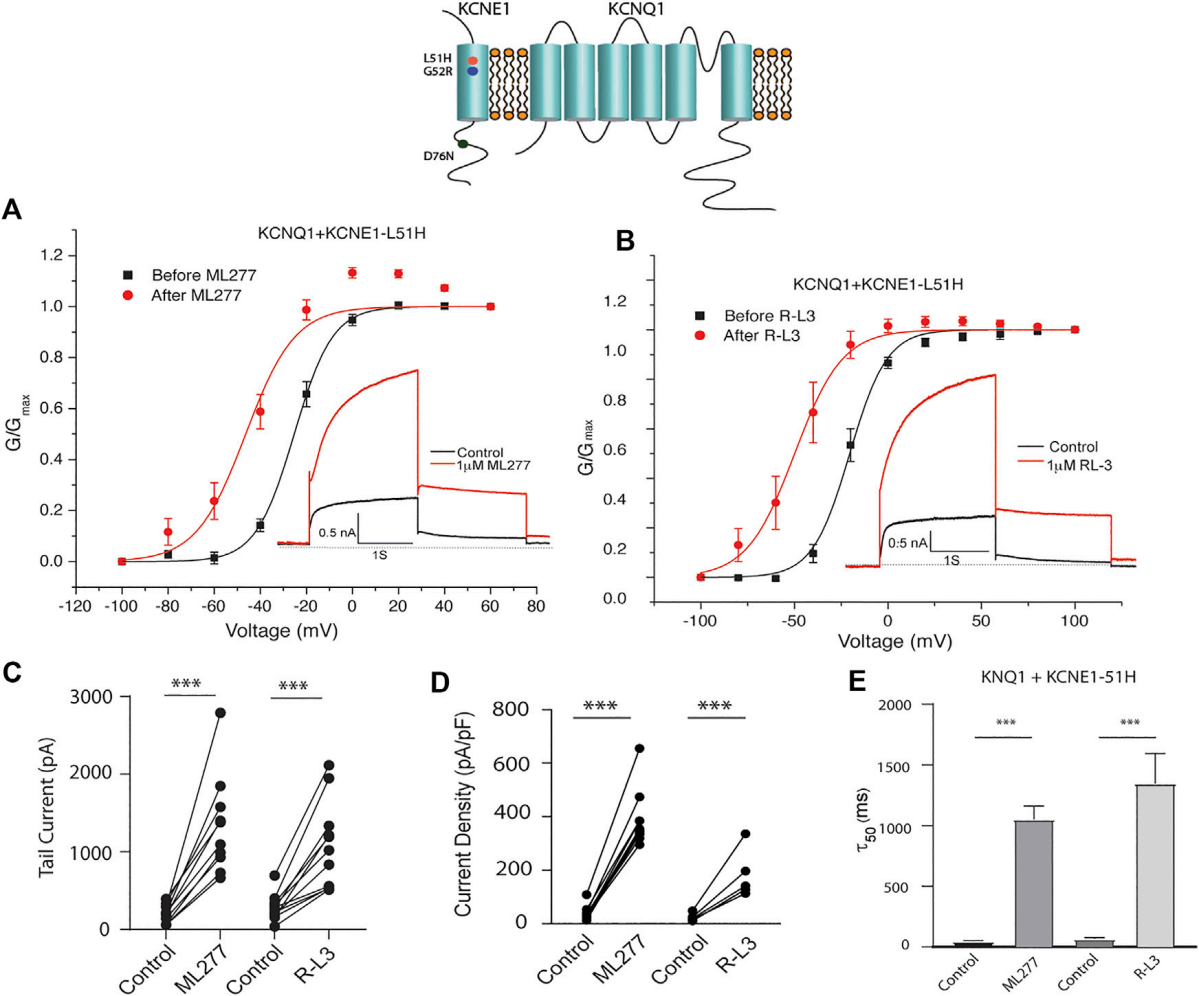
Potentiation of KCNQ1-L51H channel activity by ML277 and R-L3. **(A)**. Normalized conductance/voltage relationship for KCNQ1/KCNE1-L51H channels before (black traces) and after (red traces) application of 1 μM ML277. The inset shows representative currents recorded at +60 mV. **(B)**. Normalized conductance/voltage relationship for KCNQ1/KCNE1-L51H channels before (black traces) and after (red traces) application of 1 μM R-L3. The inset shows representative currents recorded at +60 mV. **(C)**. Tail current amplitudes measured at −40 mV in control and 1 μM ML277 and 1 μM R-L3 solutions. **(D)**. Summary data for KCNQ1/KCNE1-L51H peak current density measured 2 s at +60 mV in the absence and presence of 1 μM ML277 and 1 μM R-L3. Data in each cell are connected by straight lines. **(E)**. Summary of measurement of slow deactivation time constant (τ50) in the absence and presence of both drugs. ML277: *n* = 10; R-L3: *n* = 9.

**FIGURE 5:**
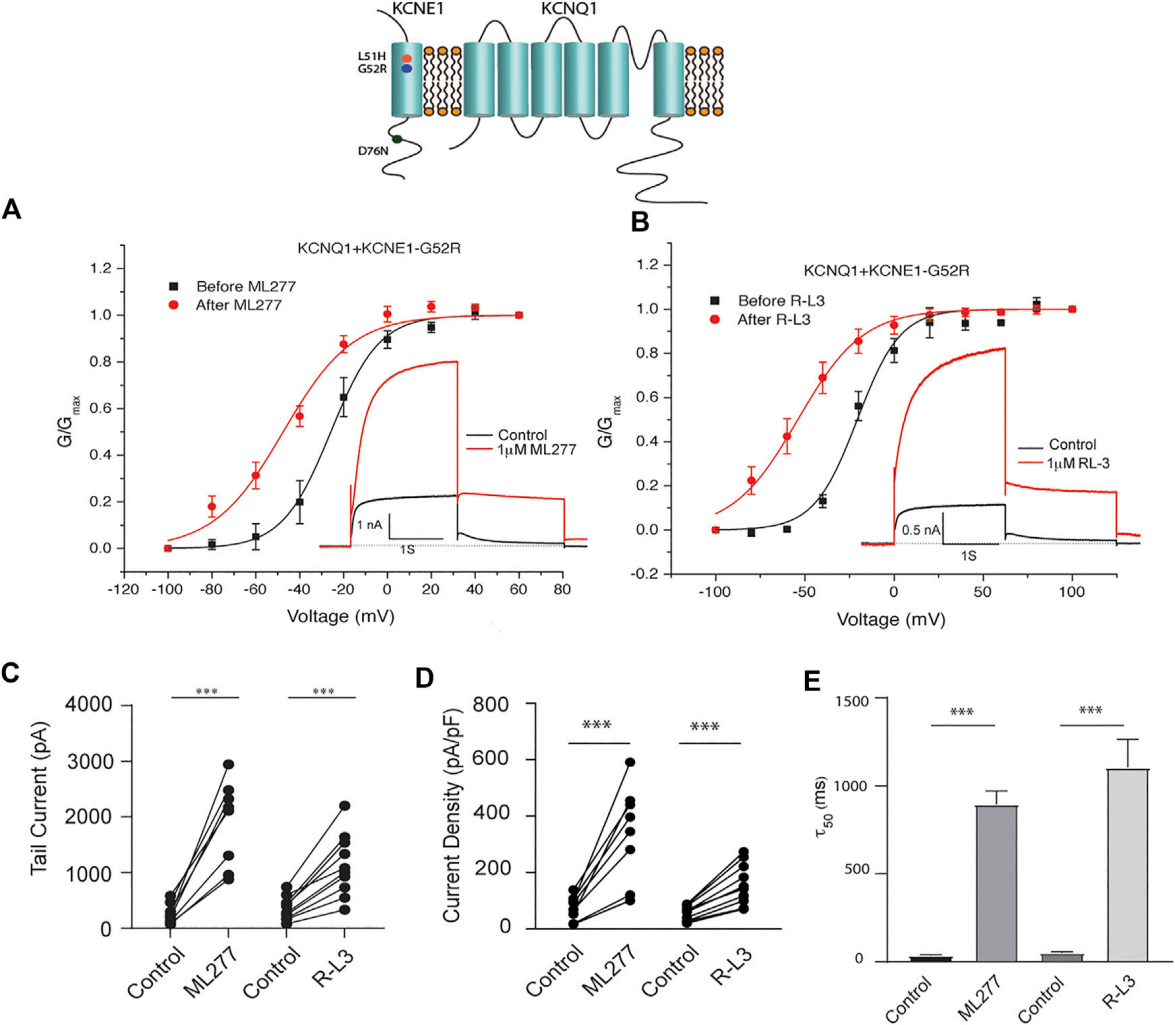
Potentiation of KCNQ1- G52R channel activity by ML277 and R-L3. **(A)**. Normalized conductance/voltage relationship for KCNQ1-KCNE1-G52R channels before (black traces) and after (red traces) application of 1 μM ML277. The inset shows representative currents recorded at +60 mV. **(B)**. Normalized conductance/voltage relationship for KCNQ1-KCNE1-G52R channels before (black traces) and after (red traces) application of 1 μM R-L3. The inset shows representative currents recorded at +60 mV. **(C)**. Deactivation of tail current amplitude measured at −40 mV in control and 1 μM ML277 and 1 μM R-L3 solutions. **(D)**. Summary data for peak KCNQ1/KCNE1-G52R channel activity measured 2 s at +60 mV in control and 1 μM ML277 and 1 μM R-L3 solutions. The currents measured in each cell are connected by straight lines. **(E)**. Summary of slow deactivation time constant (τ50) measured at −40 mV in the absence and presence of 1 μM solutions of both drugs: ML-277: *n* = 6; R-L3: *n* = 10, R-L3.

LQT5 mutations in KCNE1 lead to channel dysfunction through a multitude of pathways that result in a reduction in I_KS_ channel activity (Bohnen et al., 2017). Two LQT5 mutations, KCNE1-L51H (Bianchi et al., 1999) and KCNE1-G52R (Ma et al., 2003), that have been reported to alter intra KCNQ1-KCNE1 channel complex interactions, were utilized here to test our hypothesis that LQT5 mutations that cause I_KS_ channel activity that resembles KCNQ1 current recorded in the absence of KCNE1 might nevertheless respond to these drugs. First we tested the sensitivity of channels in which we expressed KCNQ1 and another LQT-5 mutant, D76N which has been reported in several studies to markedly reduce expressed I_KS_ channel activity (Wang and Goldstein, 1995; Splawski et al., 1997; Sesti and Goldstein, 1998; Bianchi et al., 1999). As shown in Figure 3, expression of the KCNE1 mutant D76N with KCNQ1 inhibits the agonist activity of both ML277 and R-L3, similar to expression of KCNQ1 with wild type KCNE1. We then applied 1 μM ML277 to cells co-transfected with KCNQ1/KCNE1-L51H along with a GFP marker. Here, in contrast with the insensitivity of KCNQ1/KCNE1 (WT) and KCNQ1-D76N channels to ML277, we found that ML277 increased KCNQ1/KCNE1-L51H currents, shifted the conductance voltage curve in the negative direction, and slowed deactivation of channel activity (Figure 4). Application of RL-3 led to a similar potentiation of channel current and modulation of activation and channel deactivation when KCNQ1 was expressed with L51H-KCNE1 subunits (Figure 4). We also tested another KCNE1 mutation, KCNE1-G52R, an LQT5 mutation that confers a similar current phenotype when expressed with KCNQ1 (Ma et al., 2003). Like expression with the KCNE1-L51H mutant, ML277 and R-L3 both significantly increased KCNQ1/KCNE1-G52R current amplitude, slowed channel deactivation, and caused a hyperpolarizing shift in channel activation (Figure 5), Note that in the absence of drugs, the expressed channels have reduced current amplitude, but the activation and deactivation kinetics differ from KCNQ1 alone, like altered channel activity reported by Ma, et al. and Bianchi (Bianchi et al., 1999; Ma et al., 2003) (see Supplementary Figure S1). Both drugs have marked effects on expressed channels: an increase in current amplitude, shift in activation voltage dependence and an increase in the time course of channel deactivation.

### Heterozygous LQT5 mutant channel current is potentiated by KCNQ1 agonists

**FIGURE 6:**
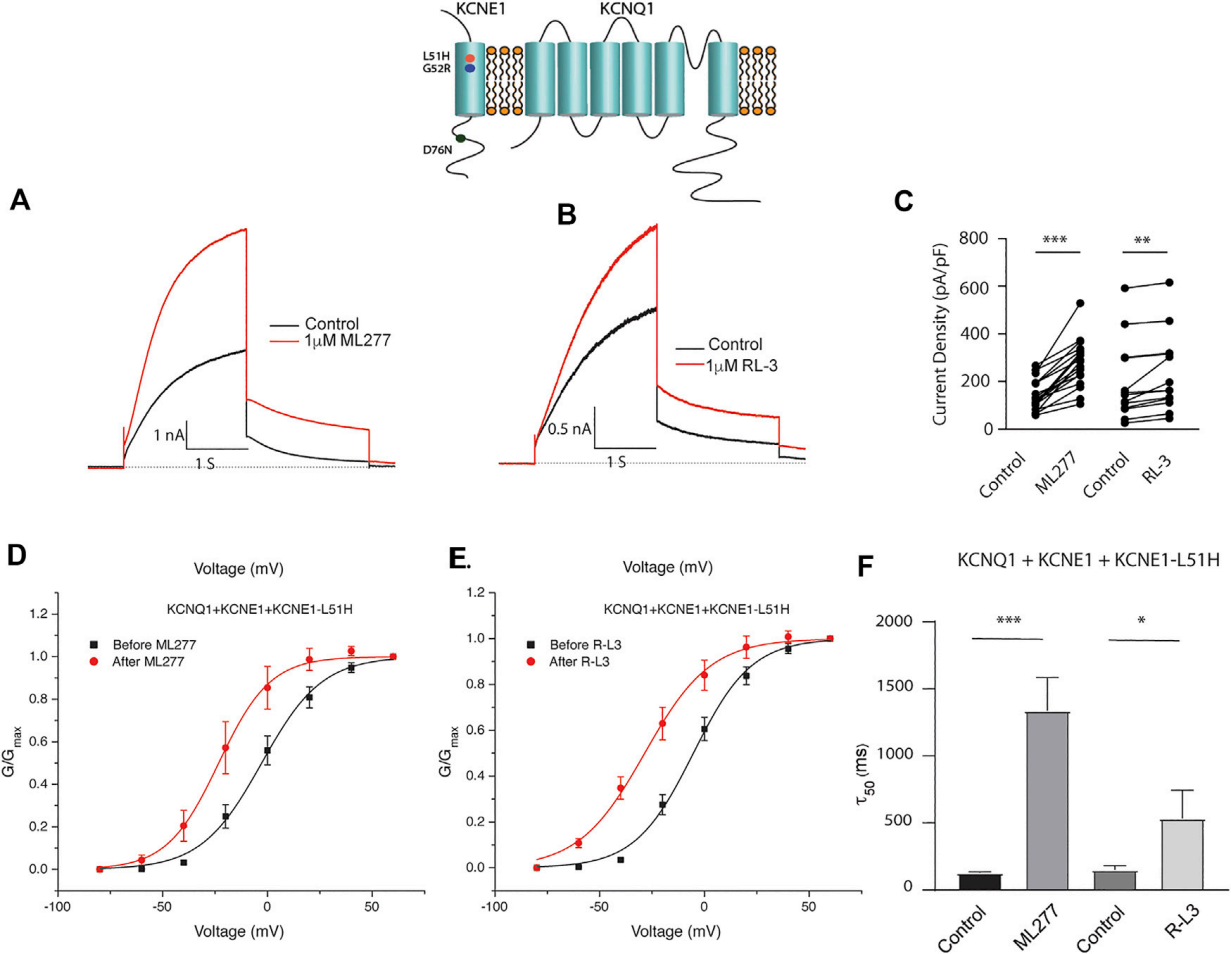
Heterozygous KCNQ1- L51H mutant channel activity is potentiated by ML277 and R-L3. Cells were transfected with KCNQ1/KCNE1/KCNE1-L51H and exposed to 1 μM ML277 **(A)** and 1 μM R-L3 **(B)**. Transfections were carried out with KCNQ1 and KCNE1 WT and KCNE1 mutant ratios of 1:0.5:0.5 as described in the text. Currents recorded at +60 mV are shown before (black traces) and after (red traces) exposure to drugs. **(C)**. Summary data, in which peak currents measured at + 60 mV at 2 s in the absence and presence of drugs are connected by straight lines. **(D)**. Normalized conductance/voltage curves measured in the absence (black) and presence (red) of 1 μM ML 277. **(E)**. Normalized conductance/voltage curves measured in the absence (black) and presence (red) of RL-3. **(F)**. Summary of slow deactivation time constant (τ50) in the absence and presence of 1 μM of both drugs. ML277: *n* = 14; R-L3: *n* = 10.

**FIGURE 7:**
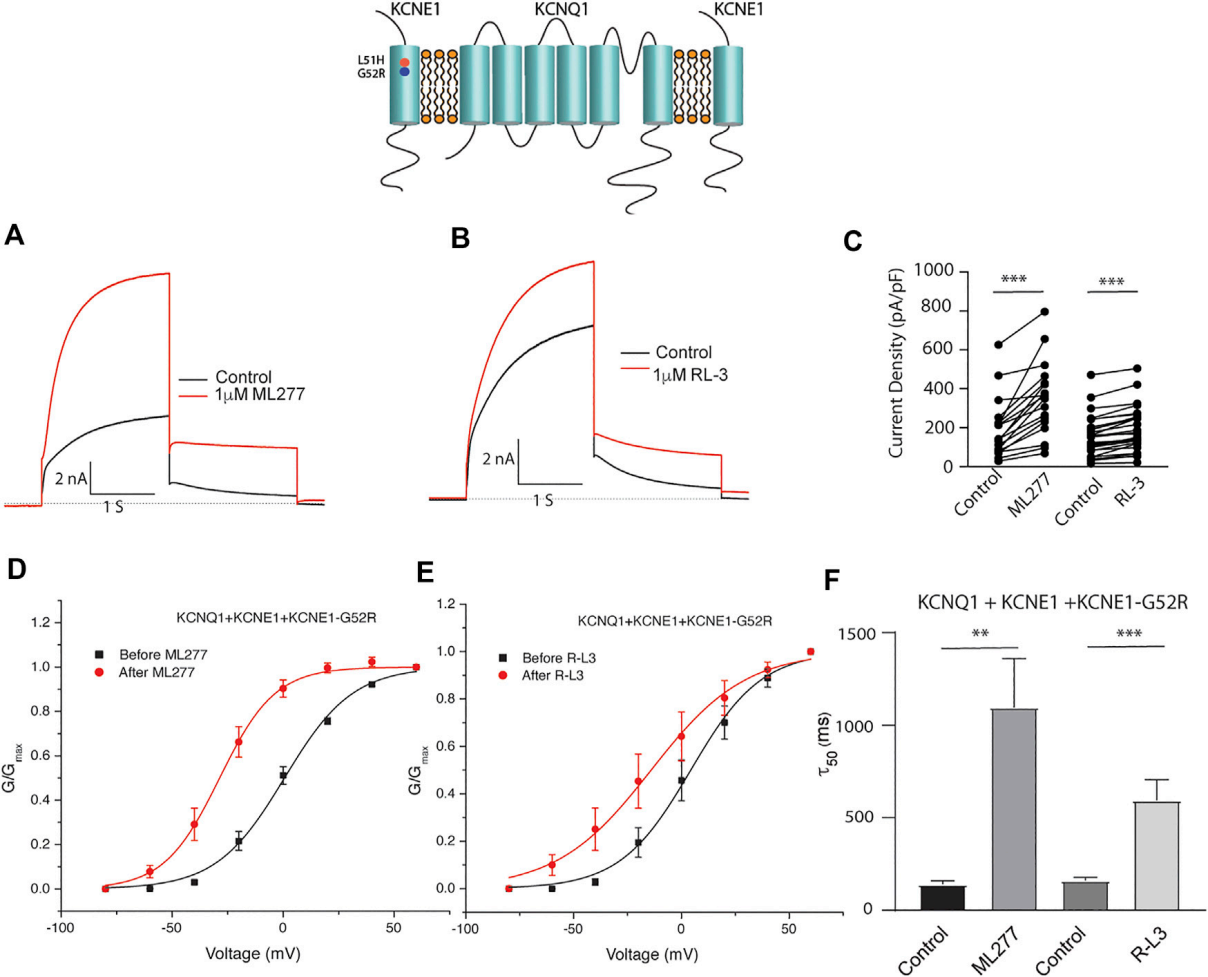
Heterozygous KCNE1-G52R mutant channel activity is potentiated by KCNQ1 agonists. Cells were transfected with KCNQ1/KCNE1/KCNE1-G52R and exposed to 1 μM ML277 **(A)** and 1 μM R-L3 **(B)**. Transfections were carried out with KCNQ1 and KCNE1 WT and KCNE1 mutant ratios of 1:0.5:0.5 as described in the text. Currents recorded at +60 mV are shown before (black traces) and after (red traces) exposure to drugs. **(C)**. Summary data for current density measured at +60 mV at 2 s in which straight lines connect peak current in the absence and presence of drugs in individual cells. **(D)**. Normalized KCNQ1/KCNE1/KCNE1-G52R conductance/voltage curves measured in the absence (black) and presence of 1 μM ML 277 (red). **(E)**. Normalized KCNQ1/KCNE1/KCNE1-G52R conductance/voltage curves measured in the absence (black) and presence of 1 μM RL-3 (red). **(F)**. Summary of slow deactivation time tail constant (τ50) measured at −40 mV in the absence and presence of 1 μM each drug. ML277: *n* = 14; R-L3: *n* = 20.

Patients harboring these mutations are likely to have heterogenous populations of mutant and WT *β* subunits. As such, we transfected KCNQ1/KCNE1/KCNE1-L51H or KCNQ1/KCNE1/KCNE1-G52R (Ratio 1:0.5:0.5) to achieve a similar total KCNE1 concentration in a more physiologically relevant manner. Here, individual channels contain a handful of different combinations of *α* subunits relative to WT and mutant *β* subunits. Like results of experiments with homozygous expression of KCNQ1 and mutant KCNE1 subunits, 1 μM ML277 or 1 μM R-L3 increased currents and slowed deactivation (Figure 6 and 7). The magnitude of these responses was intermediate between the effects of these drugs on WT KCNE1 and mutant KCNE1 *β* subunits in homozygous experiments. The compounds act similarly, however, the magnitude of the effects is greater for ML277 in both homozygous and heterozygous preparations.

### ML277 and R-L3 rescue select LQT1 I_KS_ channel activity

**FIGURE 8:**
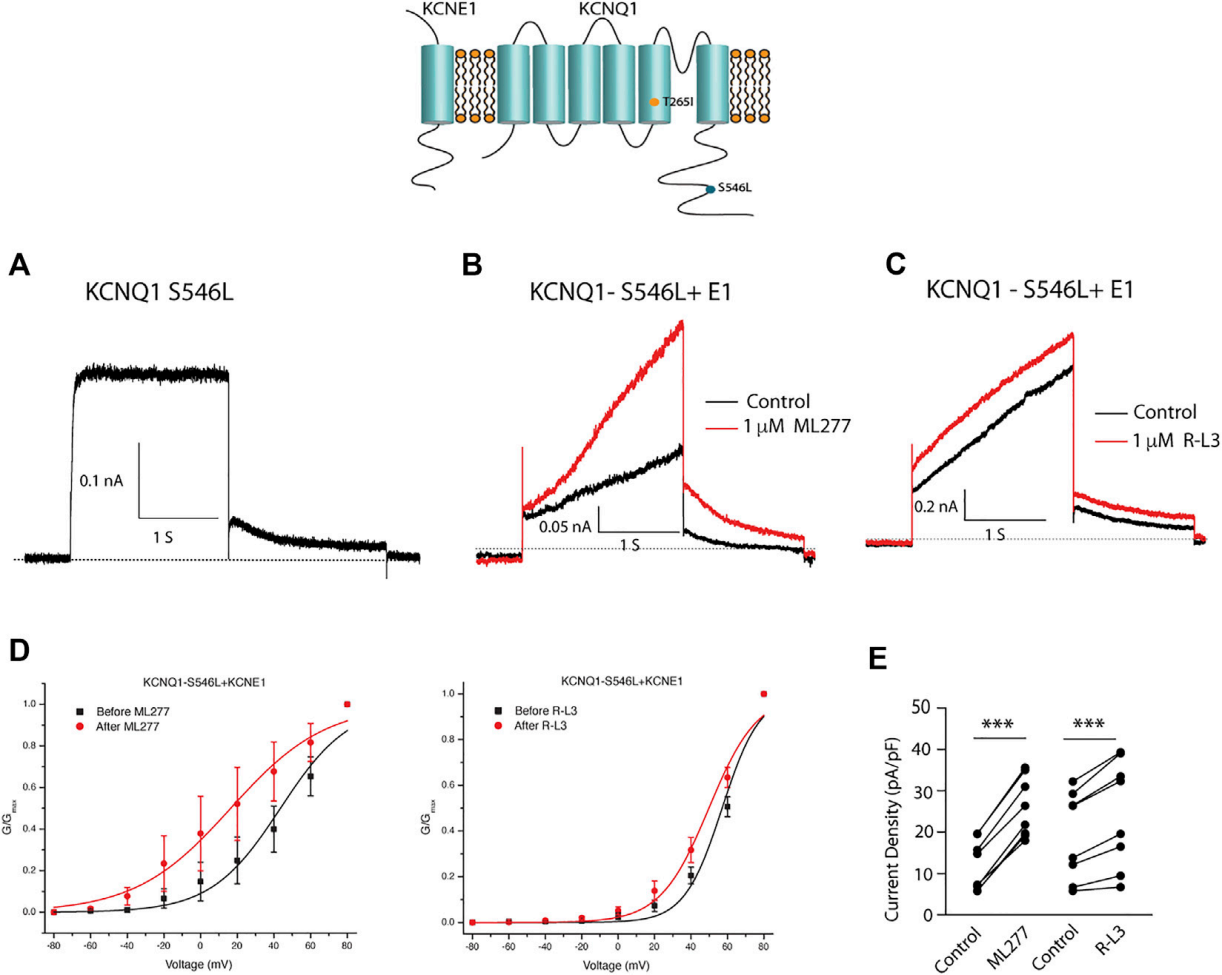
Select LQT-1 mutant channel currents are augmented by ML277 and R-L3. **(A)**. Cells were transfected with KCNQ1-S546L without KCNE1. **(B)**. KCNQ1-S546L-KCNE1 channel activity measured at +60 mV in the absence (boack) and presence (red) of 1 µM ML277. **(C)**. KCNQ1-S546L-KCNE1 channel activity measured at +60 mV in the absence (boack) and presence (red) of 1 μM R-L3. **(D)**. Normalized conductance/voltage curves measured in the absence (black curves) and presence (red curves) of 1 μM ML 277 (left panel) or RL-3 (right panel). **(E)** Summary data for peak currents measured after 2 s depolarization to +60 mV in the absence (control) and presence of 1 µM ML277 or 1 μM R-L3 with straight lines joining recordings for individual cells.

**FIGURE 9:**
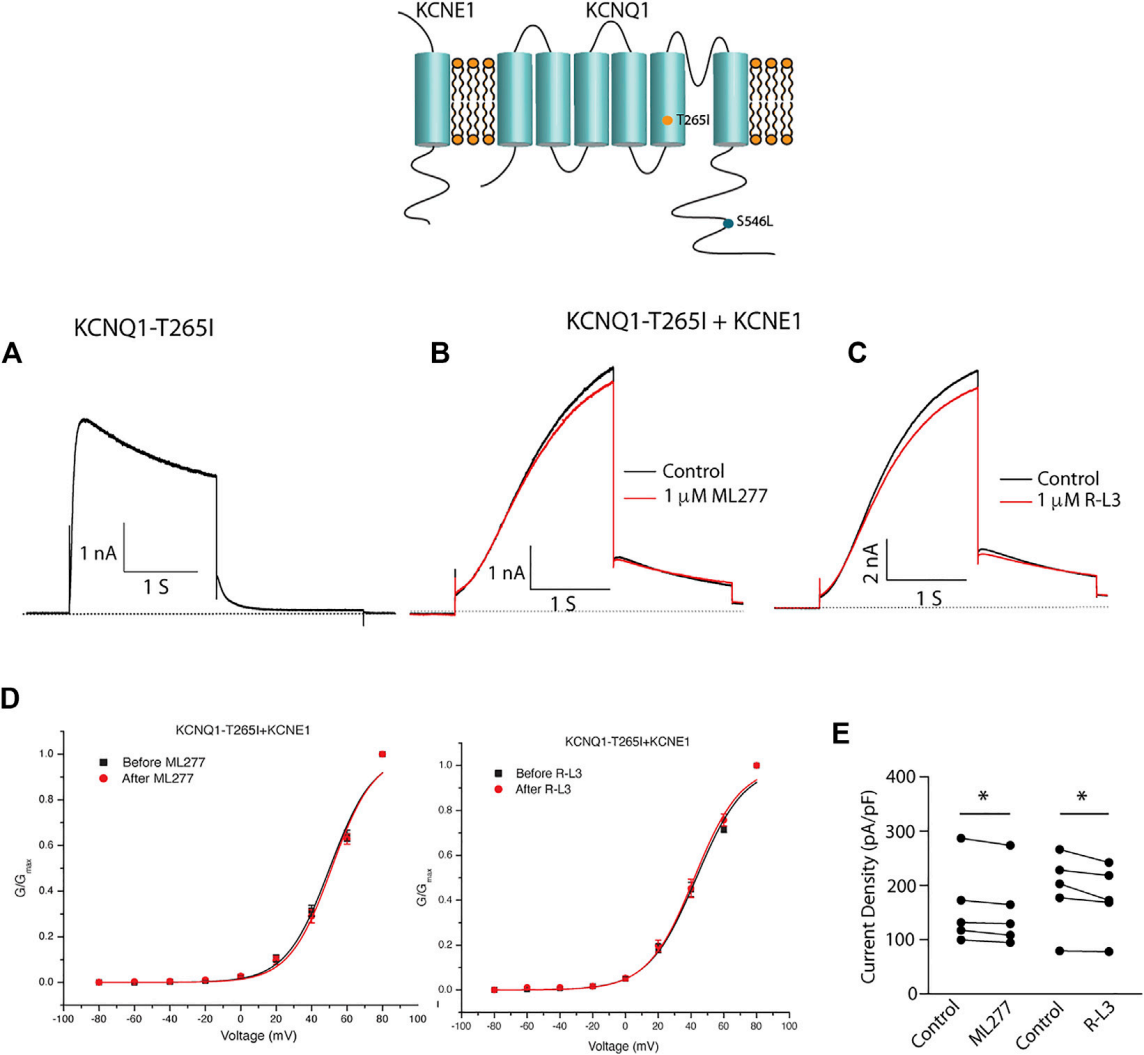
Select LQT-1 mutant channel currents are not augmented by ML277 and R-L3. **(A)**. Expression of KCNQ1-T265I without KCNE1 recorded at +60 mV. **(B)**. KCNQ1-T265I-KCNE1 channel activity at +60 mV in the absence (black trace) and presence (red trace) of 1 µM ML277. **(C)**. KCNQ1-T265I-KCNE1 channel activity at +60 mV in the absence (black trace) and presence (red trace) of 1 μM R-L3. **(D)**. Normalized KCNQ1-T265I-KCNE1 channel conductance/voltage curves measured in the absence (black) and presence of 1 μM ML277 (red curve, left panel) or RL-3 (red curve right panel). **(E)**. Summary data for peak current density measured after 2 s depolarization to +60 mV in the absence (control) and presence of 1 μM of each drug with straight lines joining recordings for individual cells.

We next tested the hypothesis that mutations in KCNQ1 that also alter the assembly of KCNQ1 with KCNE1 would yield channels that could be enhanced by ML277 and/or R-L3. We first transfected cells with the LQT1 mutant KCNQ1-S546L which had previously been shown to promote a weaker interaction of KCNE1 and KCNQ1 in the assembly of I_KS_ channels. We expressed this mutant channel with and without KCNE1 (Dvir et al., 2014). We found that the expressed channel activity in the presence of KCNE1 was enhanced by both drugs (Figure 8) providing evidence that mutation-induced alteration of KCNE1 and KCNQ1 assembly underlies agonist activity of both compounds. To further support the hypothesis, we next tested the sensitivity of currents in cells transfected with a different LQT1 mutant, KCNQ1-T265I with and without KCNE1. This mutation had previously been shown to alter expressed channel kinetics but not to disrupt subunit assembly (Yang et al., 2009). Here we found that ML277 or R-L3 did not enhance I_KS_ current expressed in these cells (Figure 9) supporting our hypothesis that it is mutation-induced alteration of KCNQ1/KCNE1 interactions that underlie agonist activity of both compounds.

### Assembly of KCNQ1 and KCNE1 subunits

**FIGURE 10:**
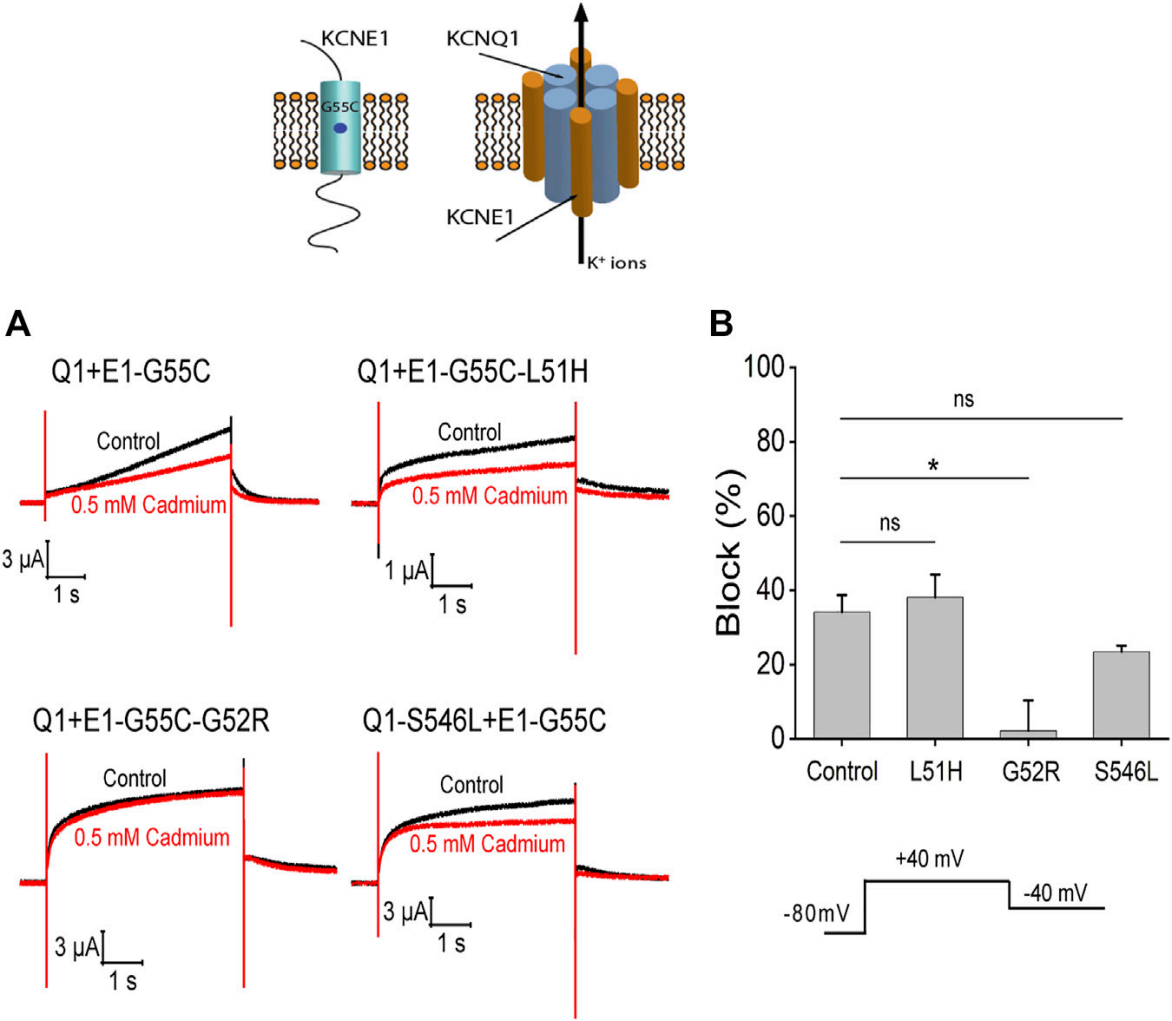
Cd^2+^ sensitivity of KCNQ1 and KCNE1 mutants. **(A)** Currents in response to voltage step to +40 mV from −80 mV with a tail voltage of −40 mV in the absence (control) and presence of 0.5 mM cadmium. **(B)** Percent block of 0.5 mM cadmium for each mutation (*n* > 3). Significance tested by ANOVA with the Dunnett’s test for multiple comparisons with Control (Q1+E1-G55C). Ns = non-significant, *p < 0.05.

To test whether KCNQ1 and KCNE1 subunits assemble in the different mutations, we introduced the mutation G55C in KCNE1. It has previously been shown that the G55C mutation confers external Cd^2+^ sensitivity to KCNQ1/KCNE1-assembled channels (Tai and Goldstein, 1998; Tapper and George, 2000). We therefore expressed KCNQ1/KCNE1-G55C, KCNQ1/KCNE1-L51H/G55C, KCNQ1/KCNE1-G52R/G55C, and KCNQ1-S546L/KCNE1-G55C in *Xenopus* oocytes and recorded currents before and after application of external Cd^2+^. The application of 0.5 mM Cd^2+^ reduced the currents in response to a voltage step to +40 mV by around 50% in KCNQ1/KCNE1-G55C, KCNQ1/KCNE1- L51H/G55C, and KCNQ1-S546L/KCNE1-G55C channels (Figure 10A). There were no significant differences in the percent inhibition by Cd^2+^ of the currents in KCNQ1/KCNE1-G55C, KCNQ1/KCNE1-L51H/G55C, and KCNQ1-S546L/KCNE1-G55C channels (Figure 10B), suggesting that the KCNQ1 and KCNE1 subunits assemble in KCNQ1/KCNE1-L51H/G55C and KCNQ1-S546L/KCNE1-G55C channels. However, the fact that these channels display different kinetics, KCNE1 modulation, and ML-277 sensitivity than KCNQ1/KCNE1 channels suggest that the KCNQ1 and KCNE1 subunits in these mutations do not interact in a similar manner as in WT KCNQ1/KCNE1-G55C subunits. The application of 0.5 mM Cd^2+^ did not reduced the currents in KCNQ1/KCNE1-G52R/G55C channels (Figure 10A). The percent inhibition by Cd^2+^ of the currents in KCNQ1/KCNE1-G52R/G55C channels was significantly reduced compared to KCNQ1/KCNE1-G55C channels (*p* = 0.014) (Figure 10B), suggesting either that the KCNQ1 and KCNE1-G52R/G55C subunits do not assemble at all or do not assemble correctly as WT KCNQ1 and KCNE1 subunits to confer Cd^2+^ sensitivity on KCNQ1/KCNE1-G52R/G55C channels.

## Discussion

Both ML277 and R-L3 are KCNQ1 channel activators, that can greatly increase the amplitude of I_KS_ channel activity and thus have potential for therapeutic treatment of LQT-1 or LQT-5 patients. In this paper, we test the hypothesis that these may have great utility in an approach to treatment of patients carrying specific mutations in either of the two subunits, KCNE1 or KCNQ1, that assemble to form I_KS_ channels but that reduce expressed channel activity.

There is no general agreement on the stoichiometric ratio of KCNE1 to KCNQ1 subunits underlying I_KS_ (Morin and Kobertz, 2008; Nakajo et al., 2010; Plant et al., 2014; Murray et al., 2016), although a variable stoichiometry of 4:1 up to 4:4 is possible (Murray et al., 2016). Evidence for a variable stoichiometry has been provided by studies in stem cell-derived cardiomyocytes as well as in studies of expressed channels in heterologous expression systems (Wang et al., 1998; Wang et al., 2011; Wang and Kass, 2012; Thompson et al., 2018). Studies in hiPSC-CMs suggest that, in more physiological situations than heterologous expression systems, WT KCNE1 may not saturate the potential WT KCNQ1 binding site. For example, Wuriyanghai et al. (2018) reported expression of a novel LQT-1 mutation, KCNQ1-A344D, expressed in human induced pluripotent stem cell cardiac myocytes (hiPSC-CMs and found that ML277 exposure resulted in a significant reduction in action potential duration not only in hiPSC-CMs expressing the A344D mutation, but also in control, wild type cells. These results are like those reported by Ma et al. (2015) in which the effects of ML277 were reported to reduce action potential duration not only in cells expressing a novel KCNQ1 C-terminal deletion mutation but also in control cells that were mutation-free. Further work by Kuusela et al. (2016) indicated that ML277 shortened action potentials in iPSC-myocytes expressing the G589D C-terminal KCNQ1 mutation, the founder LQT-1 mutation of the Finnish population. These studies support the view of a range pharmacological responses in the cells studied, consistent with previous experimental evidence that expression of KCNE1 in stem cell-derived myocytes can alter biophysical channel properties in a manner suggesting that endogenous channels consist of KCNQ1/KCNE1 stoichiometry of less than 4:4 (Wang et al., 2011; Wang and Kass, 2012). The fact that I_KS_ is sensitive to ML277 and perhaps R-L3 in these studies suggest in fact that these compounds may be effective for many KCNE1 and KCNQ1 variants.

In the present study we focused on the pharmacological regulation of I_KS_ channels by ML277, which had been shown by Yu and colleagues to be inhibited by co-assembly of KCNE1 and KCNQ1 (Yu et al., 2010; Yu et al., 2013), as well as the benzodiazepine R-L3, which also had been shown to activate I_KS_ channels in a manner inhibited by expression of KCNE1 with KCNQ1 (Salata et al., 1998). We selected LQT-5 mutations, KCNE1-L51H (Bianchi et al., 1999) and KCNE1-G52R (Ma et al., 2003), that inhibit I_KS_ channel activity *via* alteration of KCNE1/KCNQ1 interactions (Bianchi et al., 2000). In addition, we studied the drug-sensitivity of the LQT-1 KCNQ1 mutation S546L that changes the interface between KCNE1 and KCNQ1 (Dvir et al., 2014) expressed with wild type KCNE1 subunits. Our results clearly show that expression of these mutant KCNE1 subunits with wild type KCNQ1 subunits or expression of wild type KCNE1 subunits with S546L mutant KCNQ1 subunits renders expressed channels sensitive to both ML277 and R-L3. Importantly we show that even in the case of modeling heterologous expression of mutant and wild type KCNE1 subunits which is likely to occur in LQT-5 patients, the channel assembly still enables ML277 to increase channel activity. A recent study has identified a location of a binding pocket for ML277 in the pore region of KCNQ1 (Chen et al., 2022) and a recent structural study has confirmed the location of this binding pocket and shown that ML277 binding in the pocket causes the drug to inhibit channel inactivation (Willegems et al., 2022) akin to co-expression with KCNE1 (Wang et al., 2020a). Taken together this suggests that co-assembly of KCNE1 and KCNQ1 precludes agonist binding to this pocket and the results of our study indicate that mutation-induced disruption of these subunit interactions can restore the agonist activity of both ML277 and R-L3. Thus, the results of our study show that a pharmacological rescue with reagents such as these, is possible for specific LQT-5 and LQT-1 mutations carried by LQTS patients.

This work was supported by NIH grant 5R01GM109763-08.

## Data Availability

The original contributions presented in the study are included in the article/Supplementary materials, further inquiries can be directed to the corresponding author.

## Supplementary material

Supplementary Figure S1 Examples of currents elicited by step depolarization to +60 mV in cells expressing WT KCNQ1 **(A)**; WT KCNQ1 expressed with WT KCNE1 **(B)**; WT KCNQ1 expressed with KCNE1-L51H **(C)**; and WT KCNQ1 expressed with KCNE1-G52R.
