# Supplementary figures and images for: Pharmacological rescue of specific long QT variants of KCNQ1/KCNE1 channels
Source: Front Physiol. 2022 Nov 23;13:902224. doi: 10.3389/fphys.2022.902224 (PMC9726718; doi:10.3389/fphys.2022.902224)

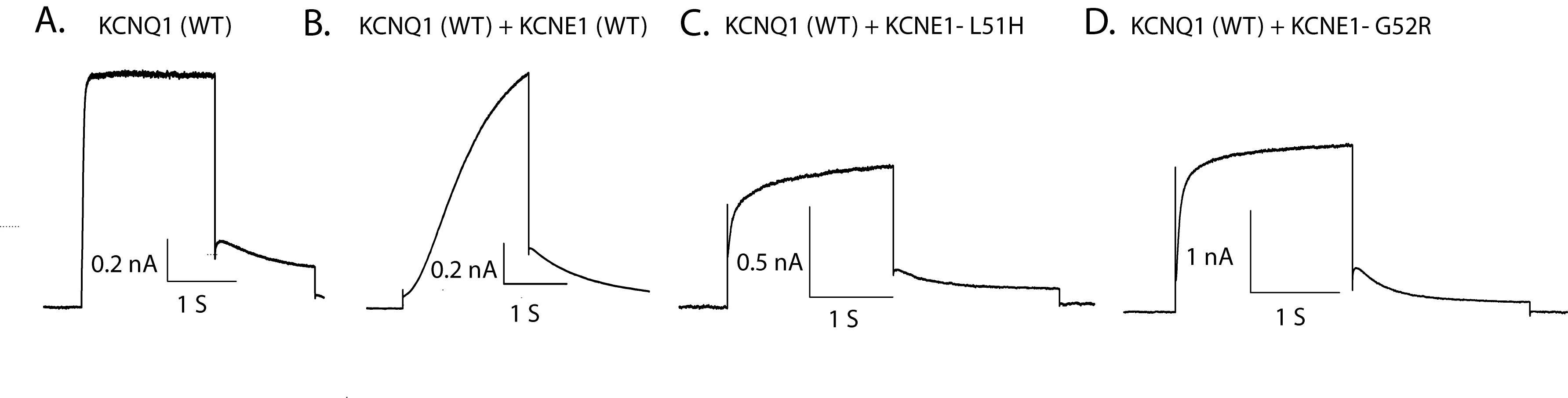

Supplement: Supplementary file 1 [file Image1.TIF]
